# Genome Sequence of Arthrobacter sp. MWB30, Isolated from a Crude Oil-Contaminated Seashore

**DOI:** 10.1128/genomeA.00013-15

**Published:** 2015-02-19

**Authors:** Jonghyun Kim, Soo Jung Kim, Seon Hee Kim, Yoon-Jung Moon, Sung-Joon Park, Seung Il Kim, Hyung-Yeel Kahng, Young-Ho Chung

**Affiliations:** a Division of Life Science, Korea Basic Science Institute (KBSI), Daejeon, South Korea; b Department of Environmental Education, Sunchon National University, Sunchon, South Korea

## Abstract

We report here the draft genome sequence of Arthrobacter sp. MWB30 strain, isolated from a crude oil-contaminated seashore in Tae-an, South Korea, which is able to degrade the crude oil and its derivatives. The draft genome sequence of 4,647,008 bp provides a resource for the identification of crude oil-degrading mechanisms in strain MWB30.

## GENOME ANNOUNCEMENT

The genus Arthrobacter was placed into the family *Micrococcaceae* by Conn and Dimmick in 1947 (1). It is an aerobic and Gram-positive bacterium with a rod-coccus morphological cycle. Bacterial species belonging to this genus typically have a DNA G+C content of 59% to 66%, and were characterized by peptidoglycan variation to the A3a (Arthrobacter globiformis group) or A4a type (Arthrobacter nicotianae group) (2, 3). These species have been found in a variety of sources, including environmentally contaminated areas such as oil, sewage, and wastewater reservoir sediments (4–7). Some of the isolates degrade oil-derivative compounds (8, 9). Recently, severe oil spill accidents have occurred in the marine environment and caused serious environmental problems. This fact stimulated us to isolate indigenous oil-degrading marine microorganisms that can be applied to the bioremediation of marine environments contaminated with petroleum oils and their derivatives. This resulted in the isolation of Arthrobacter sp. MWB30, which was capable of degrading petroleum on a seashore contaminated by crude oil after an oil spill in Tae-an, South Korea.

Strain MWB30 shared 99.99% similarity with that of the aerobic soil bacterium Arthrobacter nicotinovorans DSM 420 (X80743) based on the 16S rRNA gene sequence. Owing to the significance of strain MWB30 in bioremediation in the marine environment, its genome sequence was determined. The draft genome sequence was generated using the MiSeq 250-bp paired-end library sequencing system (Illumina Co., USA) at ChunLab, Inc. (Seoul, South Korea).

A total of 2,688,167 reads of the Illumina platform were generated and resulted in 127.15 sequencing coverages. *De novo* assembly of the sequencing reads was performed using CLCbio genomics workbench 6.0 (CLCbio, Denmark). Predicted coding genes, rRNAs, and tRNAs were archived by Glimmer 3, HMM search 2, and tRNA-scan tools, respectively. Gene annotation was accomplished by the rpsBLAST program using the NCBI COG database (http://www.ncbi.nlm.nih.gov/COG/) and the RAST server with the SEED database (10, 11). The total length of the genome was estimated to be 4,647,008 bp, assigned in 58 contigs (scaffold *N_50_*, 209,828 bp; largest contig, 599,242 bp). The G+C content was 63.0 mol%. The genome sequence of this strain included 4,465 coding sequences (CDSs), 54 tRNAs, and 2 rRNAs. *Arthrobactor* sp. MWB30 has two alkane hydroxylases, which exhibited 96% to 97% peptide sequence similarity with the alkane 1-monooxygenase of A. nicotinovorans and 94% to 98% with that of the alkane l-monooxygenase of A. aurescens TC1. Therefore, the availability of the draft genome of Arthrobacter sp. MWB30 will contribute to functional and comparative analyses for the mechanism of oil degradation.

### Nucleotide sequence accession numbers.

This whole-genome shotgun project has been deposited at DDBJ/EMBL/GenBank under the accession number JPZK00000000. The version described in this paper is version JPZK01000000.
